# Hybrid Taguchi–Gray Relation Analysis Method for Design of Metal Powder Injection-Molded Artificial Knee Joints with Optimal Powder Concentration and Volume Shrinkage

**DOI:** 10.3390/polym13060865

**Published:** 2021-03-11

**Authors:** Chao-Ming Lin, Yu-Tung Hung, Chung-Ming Tan

**Affiliations:** 1 Department of Mechanical and Energy Engineering, National Chiayi University, Chiayi 60004, Taiwan; nick87417@gmail.com; 2 Department of Mechanical Engineering, WuFeng University, Chiayi 62153, Taiwan; cmtan@wfu.edu.tw

**Keywords:** artificial knee joints, metal powder injection molding, Taguchi method, gray relation analysis, black lines, powder concentration, volume shrinkage

## Abstract

Artificial knee joints play a critical role in improving the quality of life of the elderly and those with knee injuries. Such knee joints are fabricated using a composite material consisting of metal alloy particles and polymer resin and are generally produced using the metal powder injection molding (MIM) process. However, if the local powder concentration of the molded product is too low, the mechanical properties and aesthetic appearance of the joint are severely degraded. Similarly, if the product undergoes excessive shrinkage following removal from the mold, the dimensional accuracy will fail to meet the design specifications. Accordingly, the present study applies a hybrid approach based on the Taguchi robust design methodology and gray relation analysis (GRA) theory to determine the optimal MIM processing conditions that simultaneously maximize the powder concentration uniformity while minimizing the volume shrinkage. The feasibility of the proposed approach is demonstrated by means of CAE (Computer Aided Engineering) mold flow simulations. The results show that while the robust Taguchi design method enables the optimal processing parameters that maximize the powder concentration uniformity and minimize the volume shrinkage to be individually determined, the hybrid Taguchi–GRA method enables both quality measures to be optimized simultaneously.

## 1. Introduction

**Figure 1:**
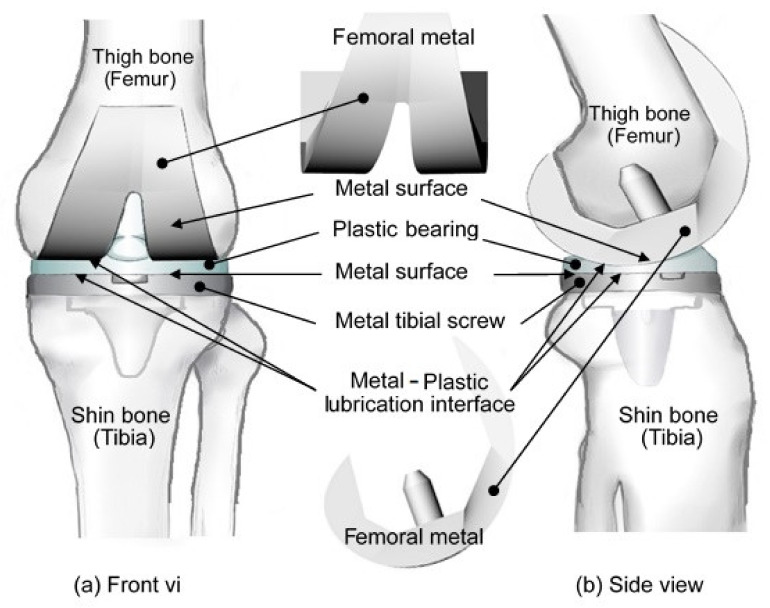
(**a**) Front view and (**b**) side view of artificial knee joint assembly, consisting of a thigh bone–femoral metal component, shin bone–metal tibial screw, and plastic insert.

The knee joint is the largest and most complex joint in the human body, connecting the ends of the femur and tibia. The joint provides an essential support pivot in standing, walking, and running. However, for those with knee joint injuries or old age, it is often necessary to replace the joint with an artificial implant in order to maintain (or restore) quality of life or mobility. The replacement procedure involves the removal of damaged bones and cartilage from the thigh, tibia, and knee bone regions and then replacing them with an artificial joint (prosthesis) made of metal alloy and polymer [1,2,3]. As shown in Figure 1, artificial knee joints consist mainly of a thigh bone–femoral metal component, a shin bone–metal tibial screw, and a plastic bearing positioned between them. The conventional materials used for artificial knee joint implantation include stainless steel, cobalt–chromium alloy, titanium, titanium alloy, tantalum, zirconium alloy, and zirconia [4]. The material library used in this study has relatively complete material information for stainless steel powder with polypropylene (abbreviated as PP), so stainless steel was used as the metal material for subsequent analysis. The relevant information for the material formula is shown in Table 1.

Artificial knee joints are extremely effective in reducing pain and restoring function in damaged or diseased knees. However, during walking, the joint experiences both a cyclic load, which causes fatigue damage, and a sliding load, which promotes wear of the plastic insert. To avoid the need for replacement surgery, the artificial joint must be capable of resisting these fatigue and wear mechanisms such that it is able to support the body weight and endure the limb rotation friction over a prolonged service life [5,6]. Accordingly, the surface mechanical properties and geometrical tolerance of the artificial knee joint components are critical concerns.

Metal injection molding (MIM) provides a rapid and convenient approach for mass production of all manner of components with complex forms, small sizes, and superior surface finish [7,8,9]. The MIM process commences by working a fine metal or ceramic powder into a plasticized polymer binder to form a feedstock material. This material is then injected into a mold in a process akin to the conventional plastic molding process. Finally, the binder is removed from the molded component by sintering technology and a sintering process is performed to improve the density and dimensional accuracy of the final component [10]. Due to the small size of the metal particles in the composite material (~10 µm), the molded components are of extremely high precision. Furthermore, the density and strength of the final sintered products are typically far higher than those of products produced using a conventional powder metallurgy process [11]. As a result, MIM products are used in a wide variety of applications nowadays, including surgical instruments, automotive components, fiber-optic parts, mobile phone parts, and biomedical implants.

However, MIM components are susceptible to significant volumetric shrinkage and warpage during the cooling process. Furthermore, an excessive shear rate during the molding process may cause the separation of the metal particles and polymer binding agent within the feedstock material, which results in turn in the formation of black lines on the surface of the molded component [12,13]. Thus, proper control of the molding parameters is essential to ensure that the finished component meets the required geometric and aesthetic standards [14,15]. In practice, the MIM processing conditions, e.g., the melt temperature, filling time, packing pressure, and so on, have both individual and interactive effects on the quality of the final molded parts. As a result, experimental trial-and-error methods for determining the optimal processing conditions are not only laborious and time-consuming, but also offer no guarantee of success. Furthermore, MIM components are typically very small, and hence discriminating between the metal powder distributions and surface defects of molded parts produced under different processing conditions is extremely challenging. Consequently, in attempting to optimize the MIM processing parameters, simulation methods tend to provide faster, more reliable, and more convenient approaches.

Among the many optimization methods available in the engineering field, e.g., performance measure modeling (PMM), response function modeling (RFM), and Taguchi robust design, the latter method provides a particularly attractive approach for optimizing the quality of manufactured products through a minimum number of experimental trials [16,17,18,19,20,21,22]. Thus, taking the metal femoral component of an artificial knee joint for illustration purposes, the present study combines the Taguchi design method with Moldex3D simulations [23,24] to determine the MIM processing conditions, which maximize the uniformity of the powder particle distribution in the final component and minimize the volume shrinkage magnitude during the cooling phase, respectively. The present study commences by performing Taguchi experiments to determine the processing conditions that optimize the uniformity of the powder concentration distribution in the final product. A second series of Taguchi experiments is then performed to establish the processing parameters that minimize the volume shrinkage of the part (i.e., the knee joint) following its removal from the mold. Finally, a gray relation analysis (GRA) technique [25] is applied to the Taguchi results to determine the processing parameters that optimize both the powder distribution and the volume shrinkage simultaneously.

## 2. Theoretical Basis for Metal Powder Injection Molding Simulation

The three basic conservation laws (mass, momentum, and energy) for generalized Newtonian fluids [26], the material constitute relationships for the specific polymer materials [27], and the particle-phase conservation relationships for a rigid spherical particle suspension in the polymer fluid [28,29,30,31] will be used as the theoretical basis for metal injection simulation analysis. In addition, this also includes the formation of black lines on the surface due to the metal particle migration effect of the mixed fluid during processing.

### 2.1. Shear-Induced Phase Separation Effect

**Figure 2:**
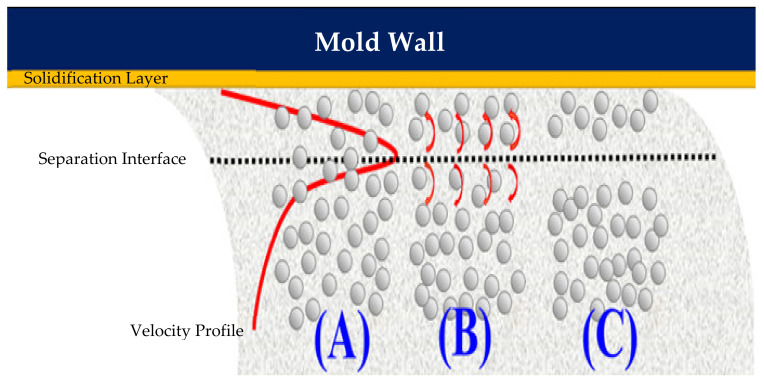
Shear-induced migration of metal powder particles in composite MIM material: (**A**) metal powder particles randomly distributed in binder matrix; (**B**) metal powder particles rotated in different directions under effects of high shear rate gradient; (**C**) phase separation of metal powder particles and binder material.

In the MIM process, the near-wall friction force produced as the molten resin flows into the mold creates a high shear rate gradient within the channel, which causes the particles to undergo local rotation (see region B in Figure 2). As the melt proceeds along the channel, the particles aligned with the point of maximum shear are pushed toward the wall or central region of the channel, respectively. As a result, a separation of the powder particles and binder material occurs (see region C in Figure 2). Since the point of maximum shear is located asymmetrically within the channel, the powder concentration has a non-uniform distribution, with a lower powder density close to the wall surface and a higher powder density toward the center of the channel [32].

### 2.2. Formation of Black Lines on Product Surface

**Figure 3:**
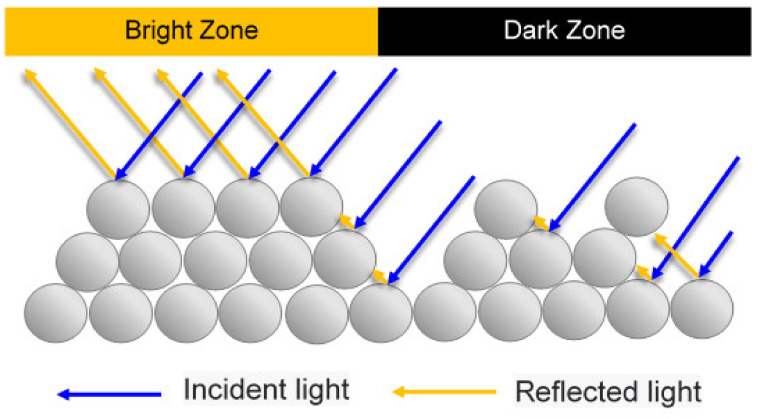
Schematic illustration showing formation of black lines under effects of non-uniform powder particle concentration distribution [11].

As shown in Figure 3, the non-uniform distribution of the powder concentration within the channel leads to a difference in behavior of the light incident on the molded part surface. In particular, in regions of the surface with a dense powder arrangement, the surface roughness is relatively lower. Consequently, the majority of the incident light is reflected from the surface, giving rise to a bright and shiny appearance. However, in the regions of the surface with a low powder concentration, some of the incident light is trapped within the voids between the particles. As a result, the reflectance of the surface is reduced and “black lines” are produced, which severely degrade the aesthetic appearance of the product.

### 2.3. Quantification of Metal Powder Concentration Distribution

In the present study, the uniformity of the powder concentration distribution is quantified by computing the standard deviation of the local powder concentration with respect to the average powder concentration in accordance with:

(1) $$\sigma =\sqrt{\frac{1}{N}\overset{N}{\underset{i=1}{\sum }}{\left(y_{i}-\mu \right)}^{2}}$$

where *y_i_* is the value of each discrete sampling point, σ is the standard deviation, *μ* is the average value, *N* is the total number of sampling points, and *i* is the sampling point index. A smaller value of Equation (1) indicates that the powder concentration has both a smaller dispersion and lies closer to the average value. In other words, the powder particles are more uniformly distributed, and hence the black line effect is reduced [33].

### 2.4. Volume Shrinkage Distribution

In practical MIM processes, the properties of the polymer material are heterogeneous rather than homogeneous and vary significantly in response to local changes in the temperature and shear rate. As a result, the melt temperature, mold temperature, packing pressure, packing time, and filling speed all have significant effects on the residual stress produced within the molded component during the filling and packing stages. In most cases, the magnitude of the residual stress varies from one region of the component to another, depending on the local geometric thickness. Consequently, the component inevitably undergoes a certain amount of warpage and shrinkage on ejection from the mold and subsequent cooling. In the present study, the extent of the volume shrinkage produced under different MIM processing conditions is again evaluated using Equation (1), where in this case, the equation is applied at all of the mesh nodes within the Moldex3D model rather than at the surface nodes only.

## 3. MIM Simulations, Taguchi Method, and Gray Relation Analysis

### 3.1. Geometry Model and Feedstock Material

As described in Section 1, the present simulations considered the metal femoral component of an artificial knee joint for illustration purposes (see Figure 1). The simulation model comprised a total of 71,290 elements and 26,035 nodes, of which 4635 of these nodes were surface nodes. The feedstock material was assumed to be CAE-MIM-001 (Supplier material number-Moldex3D), with thermophysical properties taken directly from the Moldex3D library. The material consisted of a plastic matrix (PP) and 60% metal powder (see Table 1).

### 3.2. Mold Flow Analysis

**Figure 4:**
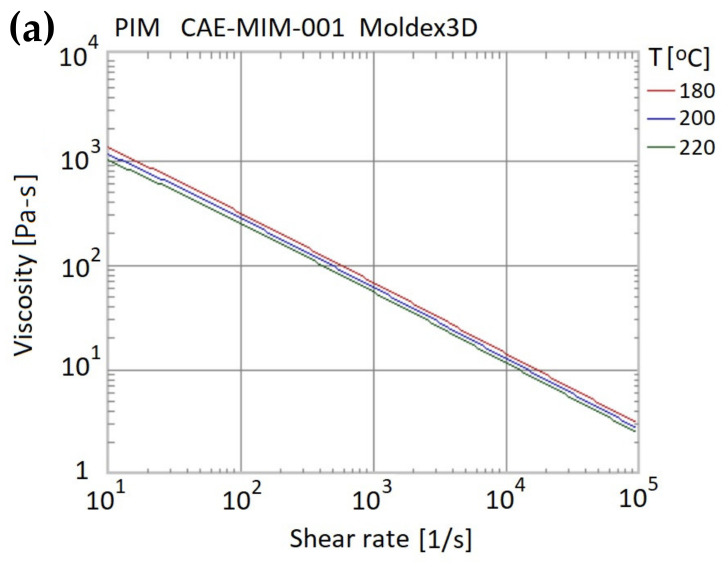

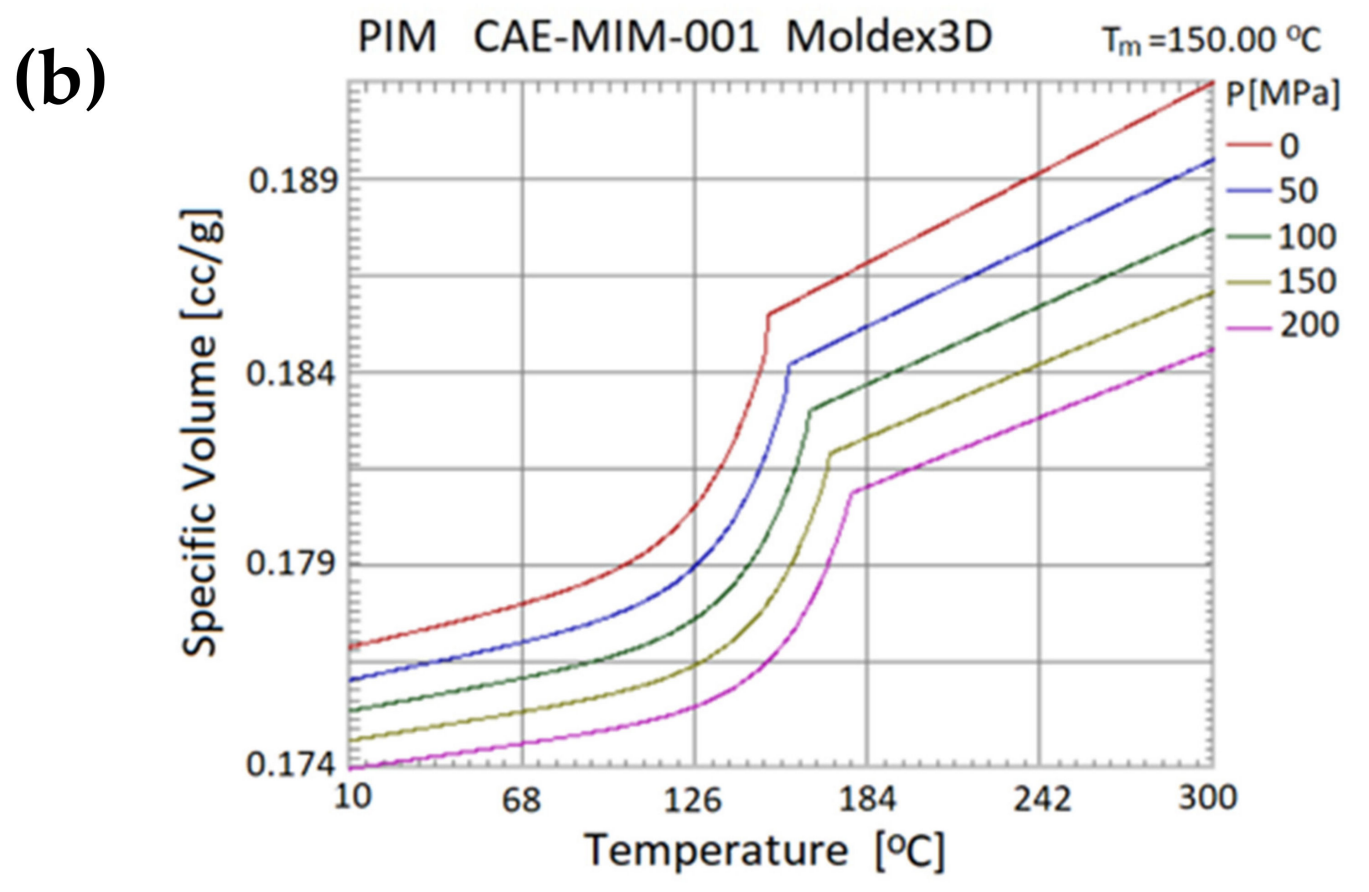
Material properties of composite material: (**a**) viscosity vs. shear rate; (**b**) specific volume vs. temperature.

**Figure 5:**
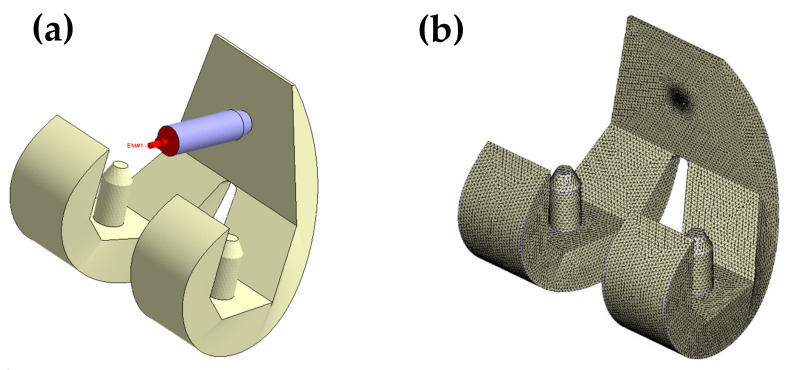
Simulation model (**a**) and mesh (**b**) of metal femoral component in artificial knee joint.

The powder particle distribution within the molded component was evaluated by means of Moldex3D CAE simulations (CoreTech System Co., Ltd., Zhubei City, Taiwan). The knee joint had the component geometry shown in Figure 1 and the thermophysical properties of the metal powder and polymer resin were assigned the values shown in Table 1. The viscosity and specific volume properties of the composite material are shown in Figure 4a,b, respectively. As shown in Figure 5, the simulation model comprised the metal femoral component of the artificial knee joint and a single flow gate. The model was constructed using boundary layer mesh (BLM) technology [34,35] and had a total volume of 41.61 cm^3^. 

### 3.3. Taguchi Experiments

**Figure 6:**
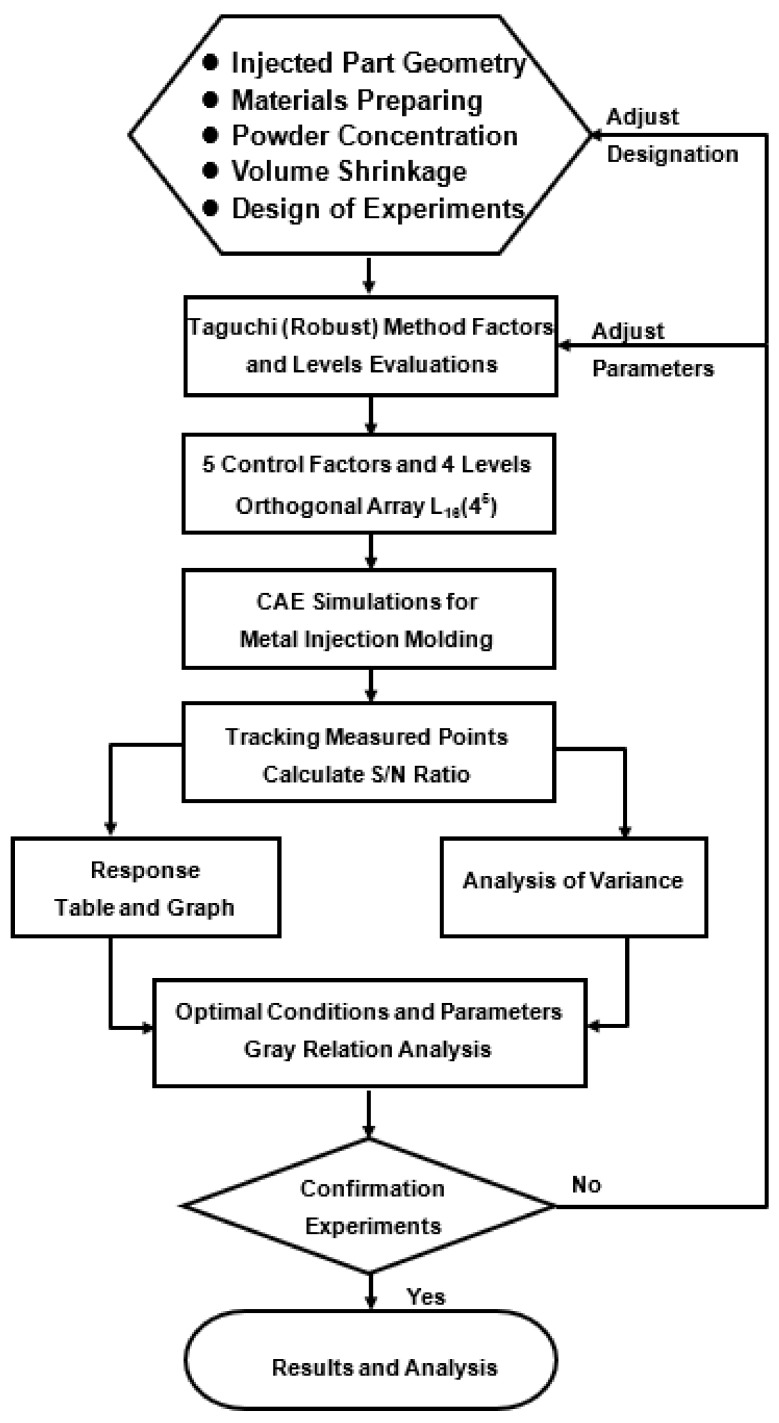
Flow chart of Taguchi–GRA framework for MIM simulations of an artificial knee joint.

In the present study, the Taguchi method was used to determine the optimal settings of the MIM processing conditions, which enhanced the powder particle concentration uniformity within the molded product and minimized the volume shrinkage. Having solved the two corresponding single-objective optimization problems, a GRA method was applied to determine the processing conditions, which jointly optimized both the powder concentration uniformity and the volume shrinkage at the same time (see Figure 6).

In the Taguchi method, the qualities of the outcomes obtained from the various runs in the orthogonal array (OA) are evaluated using a signal-to-noise (*S/N*) metric [36]. Various *S/N* ratios are applicable, depending on the particular problem under consideration. In the present study, the aim was to minimize the standard deviation metric shown in Equation (1) for both the powder particle concentration and the volume shrinkage. Consequently, the following “smaller-the-better” *S/N* ratio was employed:

(2) $$S/N=-10\log\left({\bar{y}}^{2}+S_{n}{}^{2}\right)$$

The simulations considered five control factors, namely the melt temperature (factor A), the filling time (factor B), the gate size (factor C), the mold temperature (factor D), and the packing pressure (Factor E). As shown in Table 2, each factor was assigned four levels based on the recommended processing range for the chosen MIM composite material specified in the Moldex3D-MIM software. Consequently, the experimental trials (i.e., simulation runs) were configured in an L_16_(4^5^) orthogonal array, as shown in the gray region of Table 3.

### 3.4. Gray Relation Analysis

Gray relation analysis (GRA) provides a powerful approach for solving multiobjective optimization problems characterized by multiple factors and variables with complex interrelationships between them [37,38,39]. Notably, GRA not only enables the relative importance of multiple factors in complex unknown systems to be explored, but also provides the means to identify which particular factors dominate the quality of the final outcome. In the present study, GRA was used to characterize the relationships between the *S/N* ratios obtained for the powder concentration distribution uniformity and volume shrinkage in the respective single-response Taguchi experiments in terms of a single gray relation grade. This grade was then used as the optimization target in a further Taguchi analysis to determine the control factor level settings, which jointly optimized both the powder concentration and the volume shrinkage. In implementing the GRA method, the original response sequence was normalized as follows:

(3) $$x_{i}{}^{*}=\frac{x_{i}{}^{\left(0\right)}\left(k\right)-\min x_{i}{}^{\left(0\right)}\left(k\right)}{\max x_{i}{}^{\left(0\right)}\left(k\right)-\min x_{i}{}^{\left(0\right)}\left(k\right)}\;,$$

where $x_{i}{}^{*}$ is the normalized value from the data, $\min x_{i}{}^{\left(0\right)}\left(k\right)$ is the smallest value of $x_{i}{}^{\left(0\right)}\left(k\right)$, and $\max x_{i}{}^{\left(0\right)}\left(k\right)$ is the largest value of $x_{i}{}^{\left(0\right)}\left(k\right)$.

In GRA theory, a gray relation coefficient equal to unity implies that the two sequences are related. The related expressions are given as follows:

(4) $$\Delta_{ij}\left(k\right)=||x_{i}\left(k\right)-x_{j}\left(k\right)||,$$

(5) $$\gamma \left(x_{i}\left(k\right),\;\;x_{j}\left(k\right)\right)=\frac{\Delta \min+\delta \Delta \max}{\Delta_{ij}\left(k\right)+\delta \Delta \max},$$

where *i* = 1, 2, 3, …, *m*; *j* = 1, 2, 3…, *m*; *k =* 1, 2, 3, …, *n*; Δ*_ij_*(*k*) is the absolute value difference between $x_{i}\left(k\right)$ and $x_{j}\left(k\right)$; $x_{i}\left(k\right)$ is the reference series; $x_{j}\left(k\right)$ is a specific comparison series; $\gamma \left(x_{i}\left(k\right),\;\;x_{j}\left(k\right)\right)$ is the gray relation coefficient; Δ_min_ is the smallest value; Δ_max_ is the largest value; and δ is the distinguishing coefficient, where $\delta \in \left[0,\;1\right]$. (Note that the distinguishing coefficient is an index used to distinguish among the different factors and was assigned a value of 0.5 by default in the present study.)

Having calculated all the gray relation coefficients, the corresponding gray relation grade was computed as follows:

(6) $$\Gamma \left(x_{i},\;x_{j}\right)=\frac{1}{c}\overset{c}{\underset{k=1}{\sum }}\gamma \left(x_{i}\left(k\right),\;\;x_{j}\left(k\right)\right)\;$$

where $\Gamma \left(x_{i},\;x_{j}\right)$ is the gray relation grade and *c* is the number of sequences.

The gray relation grade is used for Taguchi analysis to obtain the optimal combination of processing parameters. Figure 6 demonstrates the steps of the CAE simulation framework, Taguchi parameter design, and with gray relation analysis.

## 4. Results and Discussion

Mold flow simulations were performed for each run in the L_16_(4^5^) OA. After each run, the average value (μ) and standard deviation value (σ) of the metal powder particle concentration and volume shrinkage were computed directly from the simulation results (see blue and green regions in Table 3, respectively). Moreover, the corresponding *S/N* ratios were computed from Equation (2).

**Figure 7:**
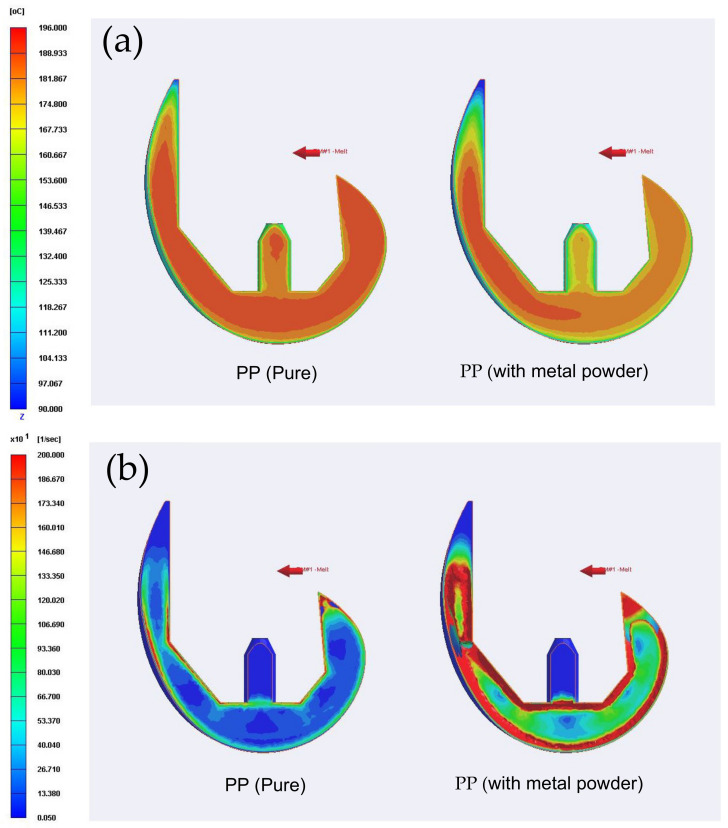
Temperature distribution (**a**) and shear rate distribution (**b**) in cross-section of artificial knee joints produced using pure polymer resin (left) and polymer resin with metal particles (right).

Figure 7 shows the simulation results obtained for the temperature distribution and shear rate distribution within artificial knee joints produced using pure polymer resin (PP (pure)) and polymer resin with metal powder (PP (with metal powder)), respectively. As shown in Figure 7a, the gate region of the component produced using polymer resin with powder particles has a lower temperature than that of the component produced using pure polymer resin due to the conductive effect of the metal particles (see Table 1). However, as shown in Figure 7b, the surface shear rate of the component produced using the composite material with metal particles is significantly higher than that of the component produced using only polymer resin. The reason is the frictional heat generated by the fluid containing metal particles flowing on the inner surface of the mold, as well as the enhanced shear effect and phase separation effect. As a result, a significant phase separation effect occurs within the composite material, giving rise to the formation of black lines on the surface of the molded component and a significant degradation of the mechanical properties of the joint. Therefore, due to the low particle concentration on the surface of artificial knee joint, the composition strength of the surface material is weak, which will not be conducive to the function of artificial knee joint that must bear wear.

### 4.1. Optimization of Powder Concentration Distribution

**Figure 8:**
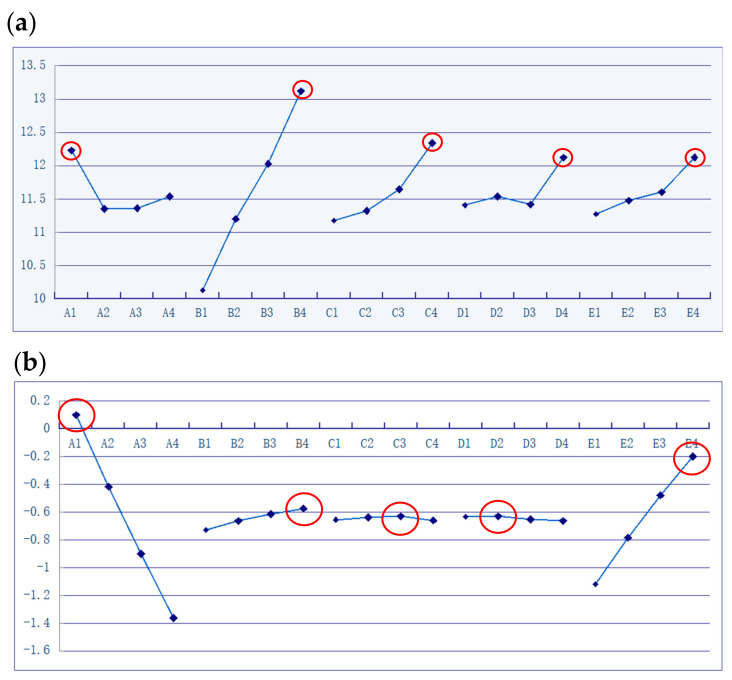
Response diagrams for powder concentration (**a**) and volume shrinkage (**b**) values (unit: dB).

In optimizing the MIM processing conditions for the powder concentration distribution, the aim was to achieve a powder concentration of 60% at each surface node. Table 3 shows the mean value (μ), standard deviation (σ), and *S/N* ratio of the powder concentration distribution for each of the 16 trials in the Taguchi OA. Table 4 and Figure 8a show the factor response analysis results for the five factors and corresponding level settings. The results show that the control factors can be ranked in order of decreasing influence on the powder concentration distribution uniformity as follows: (B) filling time, (C) gate size, (A) melt temperature, (E) packing pressure, and (D) mold temperature. In other words, a longer filling time (i.e., a slower filling velocity) reduces the shear force effect in the near-wall region of the channel, and therefore reduces the degree of phase separation (metal powder and particles) within the feedstock material. An inspection of Figure 8a shows that the optimal powder concentration uniformity is obtained using a melt temperature of 180 °C, a filling time of 2.5 s, a gate size of 7 mm, a mold temperature of 100 °C, and a packing pressure of 185 MPa (also see Table 3).

Referring to Table 3, it can be seen that the *S/N* value for this optimal trial (*S/N* = 15.455) is identical to that of the 4th trial. Hence, the robust Taguchi method is used to obtain the optimal trial by fixing filling time (most influential) factor (See Table 4). The analysis of variance shows that the filling time accounts for 70.04% of the contribution and is the largest contribution factor (see Table 4). Considering the Taguchi robust design method, a factor has a significant contribution to an objective, and this factor level is the best choice without considering the influence of this factor on other design objectives. Hence, one can fix the filling time at the optimal value of 2.5 s, redesign the orthogonal array, and conduct Taguchi analysis to obtain a set of optimal trial. The process parameters are: (A) melt temperature 180 °C, (B) filling time 2.5 s, (C) gate size 7 mm, (D) mold temperature 70 °C, (E) packing pressure 170 MPa. The results of this trial can be confirmed as the best trial by comparing them with the previous trials.

**Figure 9:**
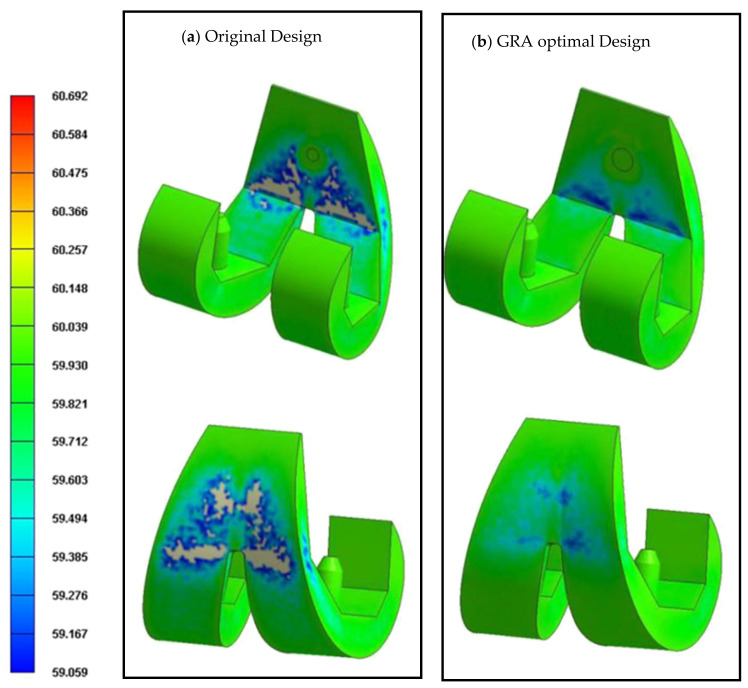
Metal powder concentration distributions in the original design (**a**) and GRA optimal design (**b**).

Figure 9 compares the powder concentration distributions of the original design and the GRA-optimized design. The results show that the hybrid Taguchi–GRA method yields an effective improvement in the uniformity of the powder concentration distribution on the part surface compared to the original design, and therefore minimizes the formation of black lines on the surface of the final component.

### 4.2. Optimization of Volume Shrinkage

**Figure 10:**
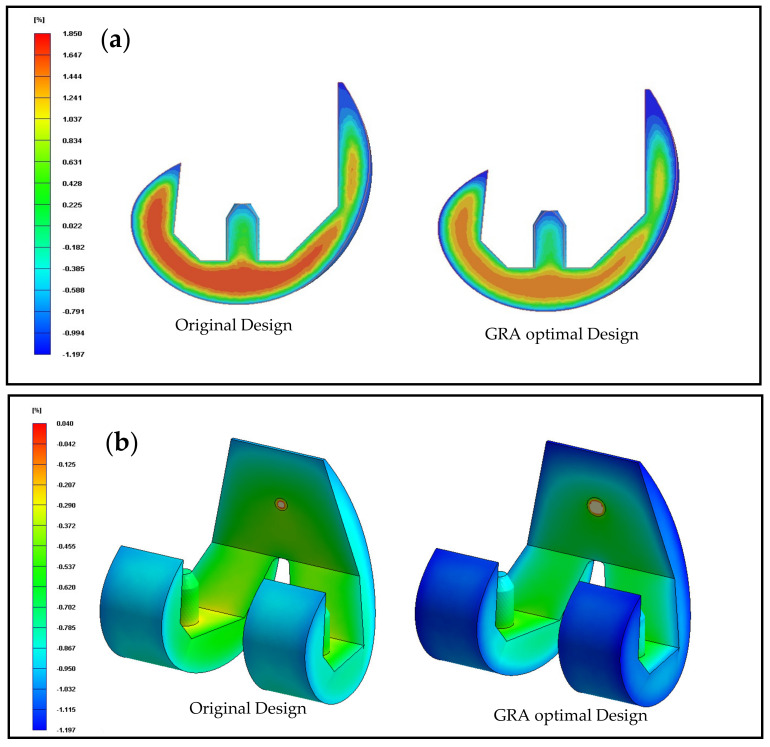
Volume shrinkage distributions in the original design (left) and GRA optimal design (right): (**a**) cross-section of the volume shrinkage distribution; (**b**) surface volume shrinkage distribution.

In determining the optimal processing conditions for the volume shrinkage of the molded component, the aim of the Taguchi experiments was to obtain volume shrinkage of 0% at each of the nodes (surface nodes and interior nodes) in the simulation model. Table 3 (green region) shows the mean and standard deviation values of the volume shrinkage obtained in each of the 16 runs in the Taguchi OA. Table 5 and Figure 8b show the corresponding factor analysis results. It is seen that the control factors can be ranked in terms of a diminishing effect on the volume shrinkage as follows: (A) melt temperature, (E) packing pressure, (B) filling time, (D) mold temperature, and (C) gate size. Moreover, the optimal process parameters are determined to be a melt temperature of 180 °C, a filling time of 2.5 s, a gate size of 6 mm, a mold temperature of 80 °C, and a packing pressure of 185 MPa. As shown in Table 5, the variance in the volume shrinkage is determined mainly by the melt temperature (contribution 70.98%) and packing pressure (contribution 28.12%). In particular, for the optimal melt temperature, the volume shrinkage reduces with an increasing packing pressure. The comparison between the original trial and the optimal trial is shown in Figure 10, which means that it has the best response to the objective function at this level, while the best level of the five control factors is set as one the processing parameters for the optimal trial. The optimal process parameters obtained are: (A) melt temperature 180 °C, (B) filling time 2.5 s, (C) gate size 6 mm, (D) mold temperature 80 °C, and (E) packing pressure 185 MPa.

Figure 10 compares the distributions of the volume shrinkage in the original design and the GRA optimized design. It can be seen that the hybrid Taguchi–GRA method yields a significant improvement in the volume shrinkage, both within the interior of the molded component (Figure 10a) and on the surface of the component (Figure 10b).

### 4.3. Multiobjective Optimization

Referring to Table 3, it can be seen that the processing conditions that optimize the powder concentration distribution uniformity are different from those that optimize the volume shrinkage. Thus, to establish the processing conditions that optimize the tradeoff between the powder concentration distribution uniformity and the volume shrinkage, a further GRA-based analysis was performed, as described below.

According to the results presented in Table 4 and Table 5, the powder concentration distribution and volume shrinkage of the molded component are dominated by the filling time and melt temperature, respectively. Accordingly, a further series of Taguchi experiments was performed, in which the filling time and melt temperature were set to their optimal values (i.e., 180 °C and 2.5 s) and the level settings of the remaining factors were varied within their respective ranges (see Table 6).

In order to perform the GRA analysis, the *S/N* data for the single-objective optimization outcomes given in Table 3 were first normalized such that the *S/N* ratios for both objective functions lay between 0 and 1. A GRA analysis was then performed using the “larger-the-better” formula. In particular, the normalized data were calculated and substituted into the GRA coefficient formula with δ set as 0.5. The GRA coefficients were then averaged to obtain the gray relation degree of the powder concentration and volume shrinkage *S/N* ratios, as shown in Table 7.

By averaging the results obtained at each level, the optimal processing conditions were found to be a melt temperature of 180 °C, a filling time of 2.5 s, a gate size of 7 mm, a mold temperature of 100 °C, and a packing pressure of 185 MPa (see Table 3). As shown in Table 8, the corresponding powder concentration *S/N* ratio was equal to 15.455 dB (i.e., 0.34% lower than that of the equivalent single-objective optimal result. Similarly, the corresponding volume shrinkage *S/N* ratio was equal to 0.582 dB (i.e., 2% lower than the equivalent single-objective optimal result).

Table 9 presents a normalized comparison of the powder concentration uniformity and volume shrinkage performance of the original design (A), single-objective optimized designs (B and C), and Taguchi–GRA design (D), respectively. Note that in compiling the table, the particle concentration distribution uniformity and volume shrinkage of the original design (A) were taken as the minimal values of the respective normalization ranges, while the optimal results obtained using the corresponding single-objective functions (B and C) were taken as the maximal values. A summed normalization value closer to 2 indicates that the corresponding design has a better ability to satisfy both design requirements simultaneously. Thus, the results presented in Table 9 confirm that the hybrid Taguchi–GRA design achieves a better overall performance than either of the two single-objective optimization designs.

### 4.4. Improvement Performance Analysis

Table 8 shows the Taguchi optimal results using the processing parameters as follows: (A) the original design, (B) the powder concentration (considering the powder concentration only), (C) the volume shrinkage (considering the volume shrinkage only), and (D) the GRA optimal design (considering both the powder concentration and the volume shrinkage simultaneously). For the single-objective optimization, considering that the two objectives cannot be optimized simultaneously, one can discuss the performance quality obtained by using the same processing conditions for another objective. In row (B), the processing parameter (the optimal powder concentration only) is used to compute the powder concentration and the volume shrinkage, whereby (μ, σ) = (59.9,0.0.134) and (μ, σ) = (0.487,0.904), respectively. In row (C), the processing parameter (the optimal volume shrinkage only) is used in computing the powder concentration and the volume shrinkage, whereby (μ, σ) = (59.864, 0.173) and (μ, σ) = (0.253, 0.899), respectively. In the above situation, it is really impossible to achieve optimal results for both objectives.

**Figure 11:**
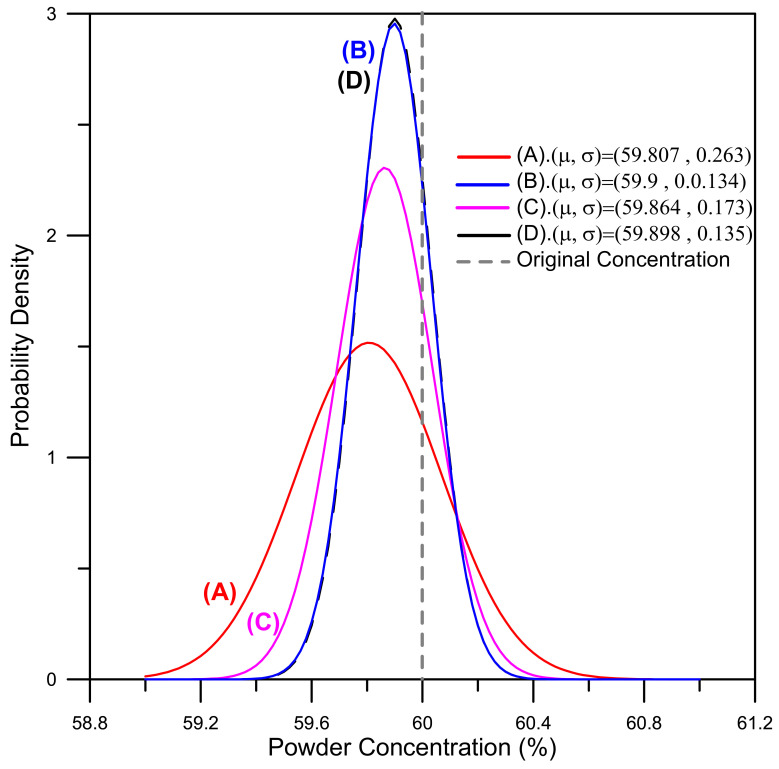
Comparison of metal powder concentration distributions in the (**A**) original design, (**B**) powder concentration optimal design, (**C**) volume shrinkage optimal design, and (**D**) GRA optimal design. (μ and σ are the average value and standard deviation of the powder concentration, respectively).

**Figure 12:**
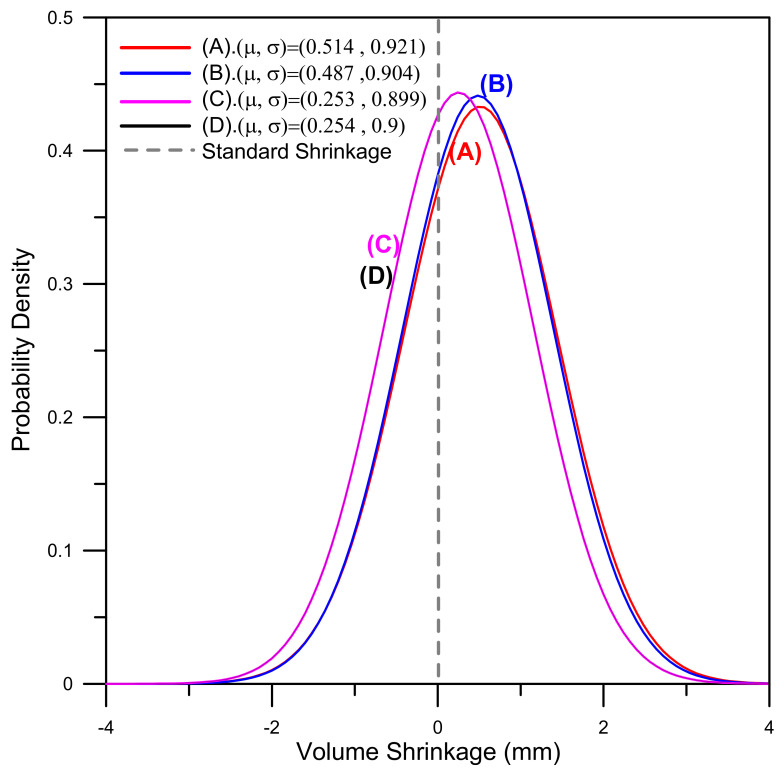
Comparison of volume shrinkage distributions in the (**A**) original design, (**B**) powder concentration optimal design, (**C**) volume shrinkage optimal design, and (**D**) GRA optimal design. (μ and σ are the average value and standard deviation of the volume shrinkage, respectively).

Referring to Table 8 once again, it can be seen that the hybrid Taguchi–GRA method improves the *S/N* for the powder concentration distribution uniformity from 9.73 dB (original design) to 15.46 (dB) and improves the *S/N* for the volume shrinkage from −0.46 dB (original design) to 0.58 (dB). Similarly, the mean powder concentration is increased from 59.807% to 59.898% (where 60% is the objective value) and the volume shrinkage is reduced from 0.514% to 0.254% (where 0% is the objective value). Finally, the standard deviations of the powder concentration and volume shrinkage are reduced from 0.263% to 0.135% and 0.921% to 0.9%, respectively. Figure 11 compares the powder concentration distributions of the original design (A), two single-objective function designs (B and C), and Taguchi–GRA design (D), respectively. It can be seen that the powder concentration single-objective design (B) and Taguchi–GRA design (D) both approach the objective value of 60% and show only slight standard deviation. Figure 12 shows the equivalent results for the volume shrinkage. It can be seen that volume shrinkage values for the single-objective design (C) and Taguchi–GRA design (D) both approach the objective value of 0% and show only slight standard deviation. Overall, the results presented in Figure 11 and Figure 12 show that the hybrid Taguchi–GRA method achieves effective improvements of both the powder concentration distribution and the volume shrinkage of the molded component, with only marginal degradation of the two quality outcomes compared to the results achieved using the respective single-objective optimization functions.

## 5. Conclusions

This study employed a hybrid Taguchi–GRA method to optimize the MIM processing conditions for an artificial knee joint in such a way as to improve both the powder concentration distribution uniformity and the volume shrinkage of the molded component. In the proposed method, single-objective Taguchi experiments are first performed to determine the optimal settings for the melt temperature, filling time, gate size, mold temperature, and packing pressure, resulting in a powder concentration uniformity close to the optimal value of 60% (i.e., the volume fraction of metal powder particles within the original feedstock material). A second series of Taguchi experiments is then performed to establish the MIM processing conditions that yield the volume shrinkage result closest to the optimal value of 0%. Finally, the *S/N* ratio results obtained in the two single-objective Taguchi experiments are used in a further Taguchi–GRA optimization process to determine the factor level settings that optimize the tradeoff between the powder concentration distribution uniformity and the volume shrinkage of the molded component. The main results of this study can be summarized as follows:

(1). The single-objective Taguchi experiments showed that for the artificial knee joint considered in the present study, the optimal powder concentration distribution uniformity was obtained using a melt temperature of 180 °C, a filling time of 2.5 s, a gate size of 7 mm, a mold temperature of 100 °C, and a packing pressure of 185 MPa. Conversely, the optimal volume shrinkage was obtained using a melt temperature of 180 °C, a filling time of 2.5 s, a gate size of 6 mm, a mold temperature of 80 °C, and a packing pressure of 185 MPa;

(2). The multiobjective Taguchi–GRA experiments showed that the optimal tradeoff between the powder concentration distribution uniformity and the volume shrinkage was obtained using a melt temperature of 180 °C, a filling time of 2.5 s, a gate size of 7 mm, a mold temperature of 100 °C, and a packing pressure of 185 MPa. Given these processing conditions, both quality outcomes approach their optimal values and show only slight standard deviation. In other words, the parameter settings both minimize the formation of black lines on the product surface and improve the dimensional accuracy of the molded component;

(3). Overall, the hybrid Taguchi–GRA optimization method successfully improves both the powder concentration uniformity and the volume shrinkage of the molded product, with no more than a slight degradation of the two quality outcomes compared to the results achieved using the corresponding single-factor optimization functions.

## Figures and Tables

**Table 1:** Material properties of polypropylene (PP) with metal (60% vol) and PP used in Moldex3D flow simulations.

Property	PP with Metal * (60% Vol)	PP
***ρ*: density** **[g/cm^3^]**	5.1	0.91
***µ*: viscosity** **[Pa-s]**	(3.5~5.5) × 10^2^ (T: 190–230 °C; $\dot{\gamma }$: 10–10^5^)	(6.8~9) × 10^2^ (T: 190–270 °C; $\dot{\gamma }$: 10–10^5^)
***α*: thermal expansion** **[1/K]**	8.5 × 10^−5^	1.5 × 10^−4^
***k*: heat conduction [erg/(s·cm·K)]**	8 × 10^4^	2.52 × 10^4^
***C_p_*: specific heat** **[erg/(g·K)]**	8 × 10^6^	3.1 × 107
***C_v_*: specific volume** **[cm^3^/g]**	0.183–0.201 (T: 10–300 °C; P: 0–200 MPa)	0.777–0.974 (T: 10–300 °C; P: 0–200 MPa)

Note: * Metal powder (stainless steel): density = 7.9 g/cm^3^; dimensions = 10 microns.

**Table 2:** The L_16_(4^5^) orthogonal array (OA) comprising five control factors and four levels.

L_16_(4^5^) OA	A Melt. Temp. (°C)	B Filling Time (s)	C Gate Size (mm)	D Mold Temp. (°C)	E Packing Pressure (MPa)
Level 1	180	1	4	70	170
Level 2	185	1.5	5	80	175
Level 3	190	2	6	90	180
Level 4	195	2.5	7	100	185

**Table 3:** Taguchi analysis results for powder concentration (blue region) and volume shrinkage (green region). (Gray region is Taguchi trials with orthogonal array).

Trials L_16_(4^5^)	A	B	C	D	E	Powder Concentration (%)			Volume Shrinkage (%)		
						μ	σ	*S/N*	μ	σ	*S/N*
**1**	180	1	4	70	170	59.807	0.263	9.733	0.514	0.921	−0.459
**2**	180	1.5	5	80	175	59.835	0.217	11.279	0.420	0.912	−0.034
**3**	180	2	6	90	180	59.855	0.190	12.440	0.335	0.906	0.304
**4**	180	2.5	7	100	185	59.898	0.135	15.455	0.254	0.900	0.583
**5**	185	1	5	90	185	59.820	0.266	9.865	0.381	0.931	−0.054
**6**	185	1.5	4	100	180	59.834	0.229	10.970	0.458	0.928	−0.297
**7**	185	2	7	70	175	59.855	0.201	12.120	0.530	0.921	−0.527
**8**	185	2.5	6	80	170	59.858	0.192	12.445	0.599	0.917	−0.789
**9**	190	1	6	100	175	59.837	0.261	10.246	0.642	0.940	−1.127
**10**	190	1.5	7	90	170	59.845	0.231	11.109	0.718	0.932	−1.415
**11**	190	2	4	80	185	59.850	0.211	11.749	0.483	0.932	−0.418
**12**	190	2.5	5	70	180	59.860	0.197	12.329	0.550	0.926	−0.642
**13**	195	1	7	80	180	59.857	0.255	10.664	0.673	0.944	−1.282
**14**	195	1.5	6	70	185	59.858	0.228	11.434	0.591	0.940	−0.910
**15**	195	2	5	100	170	59.858	0.214	11.808	0.807	0.932	−1.817
**16**	195	2.5	4	90	175	59.862	0.202	12.247	0.729	0.930	−1.449
**Optimal (Powder Concentration)**	**180**	**2.5**	**7**	**100**	**185**	**59.898**	**0.135**	**15.455**	-		
**Optimal (Volume Shrinkage)**	**180**	**2.5**	**6**	**80**	**185**	-			**0.253**	**0.899**	**0.594**

**Table 4:** Response and variance analysis for powder concentrations.

	Factor	A	B	C	D	E	Total
Level							
Level 1		**12.227**	10.127	11.175	11.404	11.274	
Level 2		11.350	11.198	11.320	11.534	11.473	
Level 3		11.358	12.029	11.641	11.415	11.601	
Level 4		11.539	**13.119**	**12.337**	**12.120**	**12.126**	
Range		0.877	2.992	1.162	0.716	0.852	
Rank		3	**1**	2	5	4	
Variance		0.69	**6.43**	1.07	0.46	0.53	9.18
Contribution (%)		7.51	**70.04**	11.66	5.02	5.78	100

**Table 5:** Response and variance analysis for volume shrinkage.

	A	B	C	D	E	Total
Level 1	**0.099**	−0.730	−0.656	−0.634	−1.120	
Level 2	−0.417	−0.664	−0.637	**−0.631**	−0.784	
Level 3	−0.900	−0.614	**−0.630**	−0.654	−0.479	
Level 4	−1.365	**−0.574**	−0.660	−0.665	**−0.200**	
Range	1.463	0.156	0.030	0.034	0.920	
Rank	**1**	3	5	4	2	
Variance	**1.58**	0.02	0.00	0.00	0.63	2.23
Contribution (%)	**70.98**	0.81	0.04	0.05	28.12	100

**Table 6:** Factors vs. levels used for robust analysis.

$L_{16}\left(4^{5}\right)$ OA	A Melt Temp. (°C)	B Filling Time (s)	C Gate Size (mm)	D Mold Temp. (°C)	E Pack Pressure (MPa)
Level 1	**180**	**2.5**	4	70	170
Level 2	**180**	**2.5**	5	80	175
Level 3	**180**	**2.5**	6	90	180
Level 4	**180**	**2.5**	7	100	185

**Table 7:** Normalized analysis results for single-factor Taguchi trials and GRA.

Trials	Powder Concentration	Volume Shrinkage	GRA
1	0.074474871	1	0.601846711
2	0.118743247	0.031603158	0.874320425
3	0.994559262	0.125072712	0.567226864
4	0.136785401	0.424339483	0.663060341
5	0.039340238	0.048821136	0.919051109
6	0.088170005	0.999974321	0.591716697
7	0.12264666	0.453345465	0.663746255
8	0.992915038	0.144401858	0.555414293
9	1	0.112484708	0.574840123
10	0.188646003	0.445416936	0.627464818
11	0.068970053	0.94038335	0.612955342
12	0	0.037172791	0.965399596
13	0.166884588	0.448893419	0.638342244
14	0.992915038	0.144401858	0.555414293
15	0.09549909	0	0.919815923
16	0.153565371	0.990535114	0.550242205

**Table 8:** Optimal results for powder concentration and volume shrinkage.

Taguchi Trials	Signal Noise Ratio: *S/N* (dB)	
	Powder Concentration (μ, σ) (%)	Volume Shrinkage (μ, σ) (%)
(**A**) Original Design	9.733472 (59.807, 0.263)	−0.458532346 (0.514, 0.921)
(**B**) Powder Concentration (Optimal Design/Robust)	15.508172 (59.9, 0.0.134)	−0.228890 (0.487, 0.904)
(**C**) Volume Shrinkage (Optimal Design)	13.143036 (59.864, 0.173)	0.593923262 (0.253, 0.899)
(**D**) Gray Relation Analysis (Optimal Design)	15.455095 (59.898, 0.135)	0.582665424 (0.254, 0.9)

**Table 9:** Normalized analysis results for overall performances.

Trials	Normalized		
	Powder Concentration	Volume Shrinkage	SUM
(**A**) Original Design	0	0	0
(**B**) Powder Concentration (Optimal Design/Robust)	1	0.218188154	1.218188154
(**C**) Volume Shrinkage (Optimal Design)	0.590431364	1	1.590431364
(**D**) Gray Relation Analysis (Optimal Design)	0.9908087	0.989303266	1.980111966

## Data Availability

Not applicable.
